# Ethyl 2-(4-chloro­phen­yl)-3-(3,5-dimethoxy­phen­oxy)acrylate

**DOI:** 10.1107/S1600536808039263

**Published:** 2008-11-29

**Authors:** Wu Chen, Yong-Ming Cui, Fei Pan, Dong-Sheng Xia, Qing-Fu Zeng

**Affiliations:** a Engineering Research Center for Clean Production of Textile Printing, Ministry of Education, Wuhan University of Science & Engineering, Wuhan 430073, People’s Republic of China

## Abstract

The title compound, C_19_H_19_ClO_5_, displays a dihedral angle of 74.7 (3)° between the mean planes of the 4-chloro­phenyl and phenol rings.

## Related literature

For phenyl­acetate and styrene derivatives, see: Fang *et al.* (2007 ▶); Huang *et al.* (2007 ▶); Li *et al.* (2007 ▶).
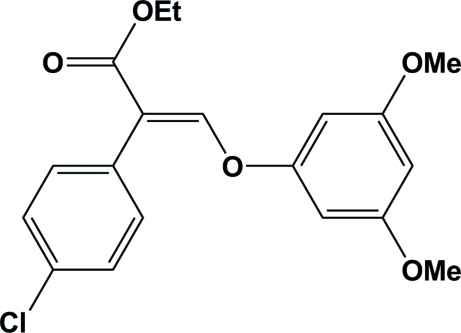


## Experimental

### 

#### Crystal data

C_19_H_19_ClO_5_
*M* *_r_* = 362.80
Triclinic, 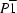

*a* = 9.601 (2) Å
*b* = 9.607 (3) Å
*c* = 10.368 (2) Å
α = 77.84 (2)°
β = 75.42 (3)°
γ = 87.40 (3)°
*V* = 904.7 (4) Å^3^
*Z* = 2
Mo *K*α radiation
μ = 0.24 mm^−1^
*T* = 298 (2) K
0.30 × 0.20 × 0.10 mm

#### Data collection

Bruker SMART 1000 CCD area-detector diffractometer
Absorption correction: multi-scan (*SADABS*; Bruker, 2001 ▶) *T*_min_ = 0.932, *T*_max_ = 0.977
6132 measured reflections
3280 independent reflections
2153 reflections with *I* > 2σ(*I*)
*R*_int_ = 0.028

#### Refinement

*R*[*F* ^2^ > 2σ(*F* ^2^)] = 0.058
*wR*(*F* ^2^) = 0.147
*S* = 1.03
3280 reflections
230 parameters
H-atom parameters constrained
Δρ_max_ = 0.48 e Å^−3^
Δρ_min_ = −0.40 e Å^−3^

### 

Data collection: *SMART* (Bruker, 2007 ▶); cell refinement: *SAINT* (Bruker, 2007 ▶); data reduction: *SAINT*; program(s) used to solve structure: *SHELXTL* (Sheldrick, 2008 ▶); program(s) used to refine structure: *SHELXTL*; molecular graphics: *SHELXTL*; software used to prepare material for publication: *SHELXTL*.

## Supplementary Material

Crystal structure: contains datablocks global, I. DOI: 10.1107/S1600536808039263/su2078sup1.cif

Structure factors: contains datablocks I. DOI: 10.1107/S1600536808039263/su2078Isup2.hkl

Additional supplementary materials:  crystallographic information; 3D view; checkCIF report

## Figures and Tables

**Fig. 1.**
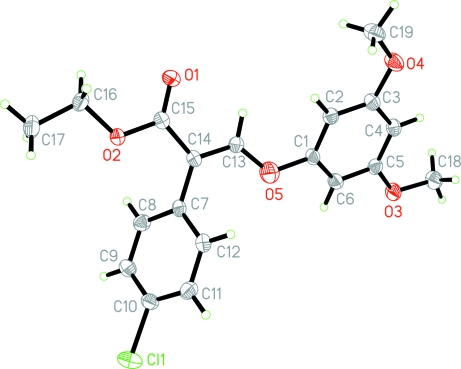
The molecular structure of compound (I), showing the atom-numbering scheme and displacement ellipsoids drawn at the 30% probabilty level.

## supplementary crystallographic information

### Comment

Phenylacetate and styrene derivatives are important for their extensive biological activities. Recently a large number of such compounds have been synthesized, and found to have good biological activities (Fang *et al.*, 2007; Huang *et al.*, 2007; Li *et al.*, 2007). Here report on the crystal structure of the new acrylate compound, (I).

The molecular structure of compound (I) is illustrated in Fig. 1. All the bond lengths and angles are within normal values. The dihedral angle between the mean plane of the 4-chlorophenyl ring (C1—C6) and the mean plane through the phenol ring (C7—C12) is 74.7 (3)°. The [O5/C13—C15/O1/O2] mean plane forms dihedral angles of 23.6 (3)° and 59.6 (3)° with the mean planes of rings (C1—C6) and (C7—C12), respectively.

In the crystal structure the molecules stack head-to-head along the c direction.

### Experimental

Ethyl 3-bromo-2-(4-chlorophenyl)acrylate (0.1 mmol) and 3,5-dimethoxyphenol (0.1 mmol) were reacted in chloroform for 12 h, giving a clear colorless solution. Crytals of compound (I) were formed by gradual evaporation of the solution.

### Refinement

All the H-atoms were placed in calculated positions and treated as rding atoms: C–H = 0.93–0.97 Å with *U*_iso_(H) = 1.2*U*_eq_(C) and 1.5*U*_eq_(methyl C).

### Crystal data

**Table:**

C_19_H_19_ClO_5_	*Z* = 2
*M**_r_* = 362.80	*F*_000_ = 382
Triclinic, *P*1	*D*_x_ = 1.335 Mg m^−^^3^
Hall symbol: -P 1	Mo *K*α radiation λ = 0.71073 Å
*a* = 9.601 (2) Å	Cell parameters from 1273 reflections
*b* = 9.607 (3) Å	θ = 2.4–25.3º
*c* = 10.368 (2) Å	µ = 0.24 mm^−^^1^
α = 77.84 (2)º	*T* = 298 (2) K
β = 75.42 (3)º	Block, colorless
γ = 87.40 (3)º	0.30 × 0.20 × 0.10 mm
*V* = 904.7 (4) Å^3^	

### Data collection

**Table:**

Bruker SMART 1000 CCD area-detector diffractometer	3280 independent reflections
Radiation source: fine-focus sealed tube	2153 reflections with *I* > 2σ(*I*)
Monochromator: graphite	*R*_int_ = 0.028
*T* = 298(2) K	θ_max_ = 25.3º
ω scans	θ_min_ = 2.1º
Absorption correction: multi-scan(SADABS; Bruker, 2001)	*h* = −11→11
*T*_min_ = 0.932, *T*_max_ = 0.977	*k* = −11→11
6132 measured reflections	*l* = −12→12

### Refinement

**Table:**

Refinement on *F*^2^	Hydrogen site location: inferred from neighbouring sites
Least-squares matrix: full	H-atom parameters constrained
*R*[*F*^2^ > 2σ(*F*^2^)] = 0.058	*w* = 1/[σ^2^(*F*_o_^2^) + (0.0584*P*)^2^ + 0.3443*P*] where *P* = (*F*_o_^2^ + 2*F*_c_^2^)/3
*wR*(*F*^2^) = 0.147	(Δ/σ)_max_ = 0.001
*S* = 1.03	Δρ_max_ = 0.48 e Å^−^^3^
3280 reflections	Δρ_min_ = −0.40 e Å^−^^3^
230 parameters	Extinction correction: SHELXTL (Sheldrick, 2008), Fc^*^=kFc[1+0.001xFc^2^λ^3^/sin(2θ)]^-1/4^
Primary atom site location: structure-invariant direct methods	Extinction coefficient: 0.015 (3)
Secondary atom site location: difference Fourier map	

### Special details

**Table:**

Geometry. All e.s.d.'s (except the e.s.d. in the dihedral angle between two l.s. planes) are estimated using the full covariance matrix. The cell e.s.d.'s are taken into account individually in the estimation of e.s.d.'s in distances, angles and torsion angles; correlations between e.s.d.'s in cell parameters are only used when they are defined by crystal symmetry. An approximate (isotropic) treatment of cell e.s.d.'s is used for estimating e.s.d.'s involving l.s. planes.
Refinement. Refinement of *F*^2^ against ALL reflections. The weighted *R*-factor *wR* and goodness of fit *S* are based on *F*^2^, conventional *R*-factors *R* are based on *F*, with *F* set to zero for negative *F*^2^. The threshold expression of *F*^2^ > σ(*F*^2^) is used only for calculating *R*-factors(gt) *etc*. and is not relevant to the choice of reflections for refinement. *R*-factors based on *F*^2^ are statistically about twice as large as those based on *F*, and *R*- factors based on ALL data will be even larger.

### Fractional atomic coordinates and isotropic or equivalent isotropic displacement parameters (Å^2^)

**Table:**

	*x*	*y*	*z*	*U*_iso_*/*U*_eq_	
C1	0.9387 (3)	0.1311 (3)	0.7046 (3)	0.0377 (7)	
C2	1.0552 (3)	0.1122 (3)	0.5995 (3)	0.0393 (7)	
H2	1.0797	0.1812	0.5197	0.047*	
C3	1.1345 (3)	−0.0115 (3)	0.6159 (3)	0.0411 (7)	
C4	1.0993 (3)	−0.1153 (3)	0.7352 (3)	0.0433 (8)	
H4	1.1517	−0.1990	0.7447	0.052*	
C5	0.9853 (3)	−0.0911 (3)	0.8389 (3)	0.0399 (7)	
C6	0.9046 (3)	0.0313 (3)	0.8241 (3)	0.0424 (7)	
H6	0.8276	0.0461	0.8947	0.051*	
C7	0.6961 (3)	0.5299 (3)	0.6619 (3)	0.0341 (7)	
C8	0.5498 (3)	0.5569 (3)	0.6821 (3)	0.0429 (7)	
H8	0.5004	0.5304	0.6245	0.051*	
C9	0.4755 (3)	0.6225 (3)	0.7858 (3)	0.0476 (8)	
H9	0.3773	0.6391	0.7982	0.057*	
C10	0.5490 (3)	0.6624 (3)	0.8697 (3)	0.0442 (8)	
C11	0.6936 (3)	0.6343 (3)	0.8557 (3)	0.0472 (8)	
H11	0.7414	0.6591	0.9154	0.057*	
C12	0.7665 (3)	0.5688 (3)	0.7519 (3)	0.0416 (7)	
H12	0.8642	0.5503	0.7417	0.050*	
C13	0.8483 (3)	0.3402 (3)	0.5672 (3)	0.0400 (7)	
H13	0.8987	0.3089	0.4896	0.048*	
C14	0.7768 (3)	0.4626 (3)	0.5486 (3)	0.0352 (7)	
C15	0.7837 (3)	0.5296 (3)	0.4060 (3)	0.0378 (7)	
C16	0.7084 (4)	0.7295 (3)	0.2597 (3)	0.0469 (8)	
H16A	0.6915	0.6667	0.2031	0.056*	
H16B	0.8017	0.7750	0.2182	0.056*	
C17	0.5928 (4)	0.8392 (4)	0.2728 (4)	0.0638 (10)	
H17A	0.5009	0.7929	0.3116	0.096*	
H17B	0.5941	0.8959	0.1844	0.096*	
H17C	0.6095	0.8993	0.3307	0.096*	
C18	1.0305 (4)	−0.3072 (3)	0.9843 (3)	0.0595 (10)	
H18A	1.0336	−0.3627	0.9167	0.089*	
H18B	0.9906	−0.3639	1.0731	0.089*	
H18C	1.1262	−0.2773	0.9788	0.089*	
C19	1.2980 (4)	0.0648 (3)	0.3998 (3)	0.0580 (9)	
H19A	1.2229	0.0832	0.3525	0.087*	
H19B	1.3822	0.0325	0.3418	0.087*	
H19C	1.3206	0.1506	0.4242	0.087*	
Cl1	0.45938 (11)	0.75361 (11)	0.99503 (10)	0.0720 (4)	
O5	0.8538 (3)	0.2570 (3)	0.6900 (3)	0.0694 (7)	
O1	0.8506 (2)	0.4862 (2)	0.3068 (2)	0.0560 (6)	
O2	0.7048 (2)	0.6494 (2)	0.39530 (19)	0.0412 (5)	
O3	0.9429 (2)	−0.1850 (2)	0.9607 (2)	0.0501 (6)	
O4	1.2510 (2)	−0.0425 (2)	0.5203 (2)	0.0545 (6)	

### Atomic displacement parameters (Å^2^)

**Table:**

	*U*^11^	*U*^22^	*U*^33^	*U*^12^	*U*^13^	*U*^23^
C1	0.0428 (17)	0.0281 (15)	0.0451 (17)	0.0087 (13)	−0.0152 (14)	−0.0104 (13)
C2	0.0505 (19)	0.0275 (15)	0.0389 (16)	0.0037 (14)	−0.0133 (14)	−0.0030 (13)
C3	0.0410 (17)	0.0355 (16)	0.0457 (18)	0.0035 (14)	−0.0090 (14)	−0.0092 (14)
C4	0.0502 (19)	0.0317 (16)	0.0469 (18)	0.0087 (14)	−0.0154 (15)	−0.0037 (14)
C5	0.0468 (18)	0.0340 (16)	0.0392 (17)	0.0037 (14)	−0.0143 (14)	−0.0047 (13)
C6	0.0441 (18)	0.0400 (17)	0.0417 (17)	0.0064 (14)	−0.0088 (14)	−0.0085 (14)
C7	0.0346 (16)	0.0272 (14)	0.0382 (16)	0.0018 (12)	−0.0096 (13)	−0.0011 (12)
C8	0.0400 (18)	0.0495 (19)	0.0417 (17)	−0.0017 (14)	−0.0125 (14)	−0.0117 (14)
C9	0.0367 (17)	0.056 (2)	0.0488 (19)	0.0084 (15)	−0.0066 (15)	−0.0145 (16)
C10	0.051 (2)	0.0403 (17)	0.0370 (17)	0.0070 (15)	−0.0037 (15)	−0.0091 (14)
C11	0.053 (2)	0.0493 (19)	0.0432 (18)	0.0025 (16)	−0.0194 (15)	−0.0101 (15)
C12	0.0383 (17)	0.0409 (17)	0.0461 (17)	0.0061 (14)	−0.0136 (14)	−0.0073 (14)
C13	0.0464 (18)	0.0365 (17)	0.0361 (16)	0.0070 (14)	−0.0114 (14)	−0.0048 (13)
C14	0.0323 (16)	0.0321 (15)	0.0404 (16)	0.0037 (13)	−0.0078 (13)	−0.0078 (13)
C15	0.0351 (16)	0.0352 (16)	0.0441 (17)	0.0025 (13)	−0.0106 (14)	−0.0097 (13)
C16	0.059 (2)	0.0425 (18)	0.0396 (17)	0.0061 (16)	−0.0195 (16)	−0.0024 (14)
C17	0.082 (3)	0.053 (2)	0.066 (2)	0.0239 (19)	−0.038 (2)	−0.0124 (18)
C18	0.074 (2)	0.0423 (19)	0.053 (2)	0.0162 (18)	−0.0132 (18)	0.0046 (16)
C19	0.063 (2)	0.046 (2)	0.051 (2)	0.0065 (17)	0.0064 (17)	−0.0061 (16)
Cl1	0.0792 (7)	0.0803 (7)	0.0582 (6)	0.0120 (5)	−0.0040 (5)	−0.0363 (5)
O5	0.0825 (19)	0.0557 (15)	0.0689 (17)	0.0162 (14)	−0.0212 (14)	−0.0105 (13)
O1	0.0676 (16)	0.0554 (14)	0.0401 (12)	0.0240 (12)	−0.0068 (11)	−0.0121 (11)
O2	0.0476 (12)	0.0376 (11)	0.0373 (11)	0.0130 (10)	−0.0115 (9)	−0.0068 (9)
O3	0.0569 (14)	0.0403 (12)	0.0454 (12)	0.0099 (11)	−0.0100 (11)	0.0031 (10)
O4	0.0587 (15)	0.0414 (13)	0.0512 (13)	0.0129 (11)	0.0011 (11)	−0.0028 (11)

### Geometric parameters (Å, °)

**Table:**

C1—C6	1.373 (4)	C12—H12	0.9300
C1—C2	1.387 (4)	C13—C14	1.338 (4)
C1—O5	1.430 (3)	C13—O5	1.366 (4)
C2—C3	1.385 (4)	C13—H13	0.9300
C2—H2	0.9300	C14—C15	1.469 (4)
C3—O4	1.363 (3)	C15—O1	1.212 (3)
C3—C4	1.392 (4)	C15—O2	1.349 (3)
C4—C5	1.378 (4)	C16—O2	1.446 (3)
C4—H4	0.9300	C16—C17	1.496 (4)
C5—O3	1.367 (3)	C16—H16A	0.9700
C5—C6	1.381 (4)	C16—H16B	0.9700
C6—H6	0.9300	C17—H17A	0.9600
C7—C8	1.388 (4)	C17—H17B	0.9600
C7—C12	1.398 (4)	C17—H17C	0.9600
C7—C14	1.487 (4)	C18—O3	1.427 (4)
C8—C9	1.385 (4)	C18—H18A	0.9600
C8—H8	0.9300	C18—H18B	0.9600
C9—C10	1.370 (4)	C18—H18C	0.9600
C9—H9	0.9300	C19—O4	1.429 (4)
C10—C11	1.379 (4)	C19—H19A	0.9600
C10—Cl1	1.745 (3)	C19—H19B	0.9600
C11—C12	1.382 (4)	C19—H19C	0.9600
C11—H11	0.9300		
			
C6—C1—C2	120.9 (3)	C14—C13—H13	117.1
C6—C1—O5	119.2 (3)	O5—C13—H13	117.1
C2—C1—O5	119.9 (3)	C13—C14—C15	115.3 (3)
C3—C2—C1	118.7 (3)	C13—C14—C7	123.7 (3)
C3—C2—H2	120.7	C15—C14—C7	121.0 (2)
C1—C2—H2	120.7	O1—C15—O2	122.0 (3)
O4—C3—C2	124.2 (3)	O1—C15—C14	126.0 (3)
O4—C3—C4	114.6 (3)	O2—C15—C14	112.0 (2)
C2—C3—C4	121.2 (3)	O2—C16—C17	107.5 (3)
C5—C4—C3	118.5 (3)	O2—C16—H16A	110.2
C5—C4—H4	120.7	C17—C16—H16A	110.2
C3—C4—H4	120.7	O2—C16—H16B	110.2
O3—C5—C4	123.3 (3)	C17—C16—H16B	110.2
O3—C5—C6	115.7 (3)	H16A—C16—H16B	108.5
C4—C5—C6	121.0 (3)	C16—C17—H17A	109.5
C1—C6—C5	119.7 (3)	C16—C17—H17B	109.5
C1—C6—H6	120.2	H17A—C17—H17B	109.5
C5—C6—H6	120.2	C16—C17—H17C	109.5
C8—C7—C12	117.6 (3)	H17A—C17—H17C	109.5
C8—C7—C14	121.8 (3)	H17B—C17—H17C	109.5
C12—C7—C14	120.6 (3)	O3—C18—H18A	109.5
C9—C8—C7	121.7 (3)	O3—C18—H18B	109.5
C9—C8—H8	119.1	H18A—C18—H18B	109.5
C7—C8—H8	119.1	O3—C18—H18C	109.5
C10—C9—C8	118.9 (3)	H18A—C18—H18C	109.5
C10—C9—H9	120.5	H18B—C18—H18C	109.5
C8—C9—H9	120.5	O4—C19—H19A	109.5
C9—C10—C11	121.4 (3)	O4—C19—H19B	109.5
C9—C10—Cl1	119.6 (2)	H19A—C19—H19B	109.5
C11—C10—Cl1	119.1 (3)	O4—C19—H19C	109.5
C10—C11—C12	119.1 (3)	H19A—C19—H19C	109.5
C10—C11—H11	120.4	H19B—C19—H19C	109.5
C12—C11—H11	120.4	C13—O5—C1	123.7 (2)
C11—C12—C7	121.2 (3)	C15—O2—C16	117.4 (2)
C11—C12—H12	119.4	C5—O3—C18	117.4 (2)
C7—C12—H12	119.4	C3—O4—C19	116.9 (2)
C14—C13—O5	125.8 (3)		
